# Existe um Tamanho Único para Todos? – Refinando a Terapia Anticoagulante Parenteral de Acordo com o Peso Corporal

**DOI:** 10.36660/abc.20250251

**Published:** 2025-05-29

**Authors:** Remo Holanda de Mendonça Furtado

**Affiliations:** 1 Brazilian Clinical Research Institute São Paulo SP Brasil Brazilian Clinical Research Institute, São Paulo, SP – Brasil; 2 Hospital Sírio Libanês Academic Research Organization São Paulo SP Brasil Academic Research Organization, Hospital Sírio Libanês, São Paulo, SP – Brasil

**Keywords:** Anticoagulantes, Peso Corporal, Heparina, Trombose

Os anticoagulantes parenterais são medicamentos amplamente utilizados na medicina. Em síndromes coronárias agudas (SCA), o uso de heparina não fracionada intravenosa (HNF) tem sido amplamente substituído por heparinas de baixo peso molecular (HBPM) devido à facilidade de administração, ao efeito mais previsível e à eficácia superior destas últimas em pacientes com infarto do miocárdio com supradesnivelamento do segmento ST submetidos à fibrinólise.^1^ As diretrizes atuais recomendam a anticoagulação com enoxaparina (classe I) em uma ampla gama de pacientes com SCA.^2,3^ No entanto, uma das principais limitações da HBPM continua sendo seu uso em pacientes com extremos de peso corporal. Alguns estudos sugerem uma anticoagulação menos confiável entre pacientes com peso superior a 100 kg ou com índice de massa corporal (IMC) acima de 40 kg/m². Ainda é controverso se a dose de 1 mg/kg a cada 12 horas para anticoagulação terapêutica deve ser mantida em pacientes extremamente obesos.^4,5^ Atualmente, a bula brasileira da enoxaparina recomenda uma dose máxima de 100 mg a cada 12 horas, com ajustes posológicos adicionais para pacientes com mais de 75 anos e aqueles com disfunção renal moderada a grave.^6^

A edição atual dos Arquivos Brasileiros de Cardiologia traz uma importante metanálise que pode ajudar, em parte, a esclarecer essas questões. Os autores^7^ realizaram uma revisão sistemática de estudos envolvendo pacientes em tratamento para diversas condições, a maioria (87%) para SCA, com anticoagulante parenteral (HNF, enoxaparina ou fondaparinux). Os estudos elegíveis compararam dois grupos de exposição (obesos versus não obesos, sendo obesos definidos como IMC ≥ 30 kg/m²) em termos de desfechos de morte e sangramento. Como poucos estudos relataram desfechos de infarto do miocárdio e nenhum analisou acidente vascular cerebral, esses desfechos não fizeram parte da metanálise atual. Os autores encontraram 6 estudos, três deles análises secundárias de ensaios clínicos randomizados (ECR) e três estudos de coorte retrospectivos. As taxas de sangramento não diferiram de acordo com a presença de obesidade (sem heterogeneidade aparente de acordo com HNF versus HBPM). No entanto, a obesidade foi associada à menor mortalidade em anticoagulação parenteral.^7^ Esta última descoberta não é totalmente surpreendente, visto que já foi demonstrada antes^8^ (o chamado "paradoxo da obesidade"). Os pontos fortes da metanálise atual foram a seleção rigorosa de estudos em diferentes bases de dados, a avaliação do risco de viés e a heterogeneidade das estimativas com métodos adequados. A principal limitação foi que, embora ECRs tenham sido incluídos, a informação essencial, ou seja, a comparação entre pacientes obesos e não obesos, foi baseada em dados observacionais, com o risco inerente de viés devido a fatores de confusão desconhecidos e não mensurados. A aparente menor mortalidade em pacientes obesos pode ser explicada pela idade geralmente mais jovem (já que o envelhecimento é, de fato, um fator que pode diminuir o peso corporal ao longo do tempo) e, portanto, pela menor prevalência de outras comorbidades relacionadas à idade que, per si, estão associadas a uma mortalidade mais alta, criando assim este aparente paradoxo. Essa mesma questão pode encobrir um sinal potencial para um aumento de sangramento (se existir) entre pacientes obesos.^9^ Se a obesidade fosse protetora contra a morte, não esperaríamos uma redução da mortalidade favorecendo a perda de peso em ECRs que testaram um medicamento para redução de peso, como a semaglutida subcutânea, entre pacientes com alto risco cardiovascular.^10^

Uma abordagem alternativa a uma metanálise sobre este tópico seria comparar estratégias de anticoagulação (por exemplo, HNF versus HBPM) entre subgrupos de IMC e realizar um teste de interação de tratamento por subgrupo para avaliar se a presença de obesidade é um modificador de efeito da estratégia de anticoagulação. Nesse sentido, estudos anteriores exploraram as potenciais interações entre peso corporal e anticoagulação parenteral. O SYNERGY foi um ECR multicêntrico, aberto, com avaliação cega de desfechos, que comparou HNF versus enoxaparina entre pacientes com SCA sem supradesnivelamento do segmento ST. A enoxaparina foi administrada na dose de 1 mg/kg a cada 12 horas, independentemente do peso corporal, sem dosagem máxima. Não houve evidência de heterogeneidade do efeito do tratamento comparando HNF versus enoxaparina de acordo com os estratos de IMC para desfechos hemorrágicos ou isquêmicos.^11^ Resultados semelhantes foram observados em uma análise conjunta dos estudos MATISSE, que randomizaram pacientes com tromboembolismo venoso agudo para fondaparinux versus HNF ou enoxaparina. A dosagem de fondaparinux foi baseada no peso corporal (com uma dose máxima de 10 mg por dia), assim como a de enoxaparina (com uma dose máxima de 100 mg a cada 12 horas). Novamente, não houve interação aparente entre os tratamentos por subgrupos nos obesos versus não obesos.^12^ Em nenhum desses estudos, o regime de anticoagulação foi adaptado com base na atividade anti-fator Xa medida no sangue.

Considerando a crescente epidemia de obesidade, a facilidade de uso de HBPM em comparação à HNF e a disponibilidade limitada de monitoramento de anti-fator Xa, a definição da melhor estratégia de anticoagulação para pacientes obesos em diversos cenários clínicos continua sendo uma questão importante e não resolvida. A metanálise atual fornece dados tranquilizadores sobre o uso seguro da anticoagulação parenteral em pacientes obesos; no entanto, futuros ECRs dedicados a essa população específica de pacientes são necessários para melhor fundamentar a prática clínica.
