# Supplementary material for: Existe um Tamanho Único para Todos? – Refinando a Terapia Anticoagulante Parenteral de Acordo com o Peso Corporal
Source: Arq Bras Cardiol. 2025 May 29;122(4):e20250251. [Article in Portuguese] doi: 10.36660/abc.20250251 (PMC12212373; doi:10.36660/abc.20250251)
Supplement: Translation

# Translation: Does One Size Fit All? – Refining Parenteral Anticoagulant Therapy According to Body Weight

**DOI:** 10.36660/abc.20250251i

**Authors:** Remo Holanda de Mendonça Furtado

**Affiliations:** 1 Brazilian Clinical Research Institute São Paulo SP Brazil Brazilian Clinical Research Institute, São Paulo, SP – Brazil; 2 Hospital Sírio Libanês Academic Research Organization São Paulo SP Brazil Academic Research Organization, Hospital Sírio Libanês, São Paulo, SP –Brazil

**Keywords:** Anticoagulants, Body Weight, Heparin, Thrombosis

Parenteral anticoagulant is widely used in medicine. In acute coronary syndromes (ACS), the use of intravenous unfractionated heparin (UFH) has been largely replaced by low molecular weight heparins (LMWH) due to the ease of administration, more predictable effect, and superior efficacy of the latter in patients with ST-elevation myocardial infarction submitted to fibrinolysis.^1^ Current guidelines recommend anticoagulation with enoxaparin (class I) in a broad range of patients with ACS.^2,3^ However, one of the major limitations of LMWH remains in its use in patients with extremes of body weight. Some studies suggest a less trustable anticoagulation among patients weighing more than 100 kilograms or with body mass index (BMI) above 40 kg/m^2^. Whether 1 mg/kg every 12 hours dosing for therapeutic anticoagulation should remain in extremely obese patients is still controversial.^4,5^ Currently, the Brazilian label from enoxaparin recommends a maximum dose of 100 mg every 12 hours, with additional dosing adjustments for patients older than 75 years and those with moderate to severe renal dysfunction.^6^

The current issue of the *Arquivos Brasileiros de Cardiologia* brings an important meta-analysis that can help, in part, to clarify these questions. The authors^7^ performed a systematic review of studies enrolling patients being treated for several different conditions, most of them (87%) for ACS, with a parenteral anticoagulant (UFH, enoxaparin, or fondaparinux). Eligible studies compared two exposure groups (obese versus non-obese, being obese defined as a BMI ≥ 30 kg/m^2^) in terms of death and bleeding outcomes. Because few studies reported endpoints of myocardial infarction and none analyzed stroke, these outcomes were not part of the current meta-analysis. Authors have found 6 studies, three of them secondary analyses from randomized clinical trials (RCT) and three retrospective cohort studies. The rates of bleeding did not differ according to the obesity status (with no apparent heterogeneity according to UFH versus LMWH). However, obesity was associated with lower mortality on parenteral anticoagulation.^7^ This last finding is not entirely surprising given it has been demonstrated before^8^ (the so called "obesity paradox"). The strengths of the current meta-analysis were the rigorous selection of studies in different databases, assessment of risk of bias, and estimates of heterogeneity with proper methods. The main limitation was that, although RCTs were included, the essential information, that is, the comparison between obese and non-obese patients, was based on observational data, with the inherent risk of bias due to unknown, unmeasured confounders. Apparent lower mortality in obese patients could be explained by the usually younger age (since aging is, in fact, a factor that may decrease body weight over time) and thus the lower prevalence of other age-related co-morbidities that *per si* are associated with higher mortality, thus creating this apparent paradox. This same issue may cover a potential signal for an increase in bleeding (if it does exist) among obese patients.^9^ If obesity were protective against death, we would not expect a mortality reduction favoring weight loss in RCTs testing a weight reduction medication, such as subcutaneous semaglutide, among patients with high cardiovascular risk.^10^

An alternative approach to a meta-analysis on this topic would be to compare anticoagulation strategies (for example, UFH versus LMWH) across BMI subgroups and test for treatment by subgroup interactions to assess whether the obesity status is an effect modifier of the anticoagulation strategy. In this regard, previous studies have explored the potential interactions between body weight and parenteral anticoagulation. The SYNERGY was a multicenter, open-label with blinded endpoint ascertainment, RCT that compared UFH versus enoxaparin among patients with non-ST elevation ACS. Enoxaparin was dosed at 1 mg/kg every 12 hours regardless of body weight without maximum dosing. There was no evidence of heterogeneity of treatment effect comparing UFH versus enoxaparin according to BMI strata for bleeding or ischemic outcomes.^11^ Similar findings were observed in a pooled analysis from the MATISSE trials, which randomized patients with acute venous thromboembolism to fondaparinux versus UFH or enoxaparin. Fondaparinux dosing was based on body weight (with a maximum dose of 10 mg per day), as was enoxaparin (with a maximum dosing of 100 mg every 12 hours). Again, no treatment by subgroup interaction was apparent for the obese versus non-obese subgroups.^12^ In none of these studies anticoagulation regimen was tailored based on anti-factor Xa activity measured in the blood.

Given the growing epidemics of obesity, the much easier use of LMWH compared to UFH, and the limited availability of anti-factor Xa monitoring, knowing the best anticoagulation strategy for obese patients in several clinical scenarios remains an important unresolved issue. The current meta-analysis provides reassuring data regarding the safe use of parenteral anticoagulation among obese patients; however, future dedicated RCTs enrolling this specific patient population are needed to better inform clinical practice.
